# Supplementary material for: TRAPID 2.0: a web application for taxonomic and functional analysis of de novo transcriptomes
Source: Nucleic Acids Res. 2021 Jul 1;49(17):e101. doi: 10.1093/nar/gkab565 (PMC8464036; doi:10.1093/nar/gkab565)
Supplement: gkab565_Supplemental_File [file gkab565_supplemental_file.pdf]

Supplementary material — TRAPID 2.0: a web application for taxonomic and functional analysis of *de novo* transcriptomes

François Bucchini[1,2], Andrea Del Cortona[1,2], Łukasz Kreft[3], Alexander Botzki[3], Michiel Van Bel[1,2] and Klaas Vandepoele[1,2,4*]

[1] Department of Plant Biotechnology and Bioinformatics, Ghent University, 9052 Ghent, Belgium

[2] Department of Plant Systems Biology, VIB, 9052 Ghent, Belgium

[3] VIB Bioinformatics Core, VIB, 9052 Ghent, Belgium

[4] Bioinformatics Institute Ghent, Ghent University, 9052 Ghent, Belgium

[*] To whom correspondence should be addressed. Tel: +32 9 3313822; Fax: +32 9 3313809; Email: klaas.vandepoele@psb.vib-ugent.be

SUPPLEMENTARY NOTES

Supplementary Note S1. Taxonomic classification evaluation

In line with other evaluation studies, taxonomic classification benchmark experiments were conducted using mock community data sets consisting of transcript sequences randomly sampled from a selection of phylogenetic clades as input, and excluding the input sequences from the reference indices.

After downloading all the RNA sequences from RefSeq (1) (release 84, September 2017), four evaluation data sets were created. Each evaluation data set consist of a collection of 10,000 transcript sequences from mock communities, where sequences encompassed in selected phylogenetic clades are randomly sampled according to predefined proportions. Similarly to other sequence databases, RefSeq suffers from sampling bias in the phylogenetic distribution of available sequences (2): two types of evaluation data sets were therefore created to take that into consideration. The first one, named 'Minimal', contains sequences sampled from a small number of well-represented phylogenetic clades, for which many RNA sequences are available within RefSeq, and favors eukaryotic clades (Supplementary Table S5). The second one, named 'Order-100', aims to be less biased and more complex in its composition, and thus contain sequences sampled from all the orders for which 100 RNA sequences or more are available (244 orders in total), in equal proportions. Two replicates of each type of data set were generated and used to perform the benchmark experiments. All taxonomic classification benchmark experiments were performed using older versions of the NCBI taxonomy and non-redundant protein database than those currently used within TRAPID 2.0, that were both downloaded on the 18th of September 2017.

A total of five classification procedures were evaluated. Kaiju was run in MEM mode (using the default minimum fragment length of 11), as well as in Greedy mode (using the default minimum score of 65), allowing up to five ('Greedy-5') amino acid substitutions/mismatches during the search, and always filtering low-complexity sequences. The same settings were replicated when Kaiju's index was split. To evaluate Kaiju's classification performance, we also executed a canonical protein sequence similarity

search with DIAMOND, as a baseline method. DIAMOND was run in 'more sensitive' mode, using a maximum E-value cutoff of 1e-5, and the taxonomic classification of each aligned input sequence was considered to be the classification of the best hit. To avoid biasing evaluation results with self-hits, proteins corresponding to sequences present in the input data set were excluded from the Kaiju and DIAMOND indices while performing the taxonomic classification benchmarks, based on simple identifier mapping. Input transcript sequences corresponding to non-coding RNAs were ignored for the evaluation.

Taxonomic classification performance was evaluated at two taxonomic ranks: phylum and genus. For each considered taxonomic rank, sensitivity and precision were calculated as outlined in (2). Sensitivity and precision values determined for each evaluation setting were subsequently averaged across data set replicates to produce single values per classification procedure and data set type (Supplementary Figure S1).

Our benchmarks confirmed the use of Kaiju for the classification of protein-coding transcripts: measured sensitivity and precision were in the range of the values reported in the evaluation reported in the original publication (figure 2 of (2), ‘454 single-end 350 nt’). Similar sensitivity and precision values were observed across the two types of input data sets (80.9-85.3% and 92.1-96.5% at phylum-level, respectively), although the classification of the ‘Order-100’ data set resulted in lower sensitivity. This difference was expected, given the paucity of closely related homologs in the reference data. For the tested benchmark data sets, DIAMOND performed better than Kaiju at the phylum level, yielding both higher sensitivity and precision, but not at the genus level where it achieved marginally better sensitivity at the cost of a lower precision than Kaiju, when considering all running modes. This observed lower precision may be due to the fact that only the top hit is taken into account to assign taxonomy. Another notable difference between the two programs is their execution time: Kaiju was reported to be 10 times faster than DIAMOND in the evaluation performed in (3). These results confirm Kaiju’s ability to successfully classify assembled transcript sequences and show that the running time required to run DIAMOND against a large reference database prohibits its use in TRAPID 2.0, where large sets of input sequences need to be processed. Comparing Kaiju running modes mirrored some of the results reported in the original publication: allowing 5 mismatches (‘Greedy-5’) resulted in better performance compared to the MEM mode at phylum-level, although in our setting the sensitivity gain was offset by a precision loss at genus-level. Finally, benchmark experiments confirmed that splitting Kaiju’s index into smaller chunks to lower its memory footprint resulted in nearly identical MEM mode performance, both at phylum and genus level. Therefore, Kaiju MEM with a split index was ultimately retained for the taxonomic classification step of TRAPID 2.0, since splitting the index did not result in a loss of performance and the MEM mode may give the best precision at genus-level, while it runs for a fraction of the time needed to perform an equivalent DIAMOND search.

Supplementary Note S2. Functional annotation evaluation

Functional annotation benchmark experiments were conducted using data from five model organisms: *Arabidopsis thaliana*, *Drosophila melanogaster*, *Escherichia coli* (strain K12), *Homo sapiens*, and *Saccharomyces cerevisiae* (strain ATCC 204508/S288c). Each organism's proteome GO annotation was retrieved from the GOA database (4) (unfiltered proteome annotation files, downloaded on 2018-11-15). The cDNA sequences corresponding to the proteins present in the annotation files were used as input for the benchmark experiments. To generate the input data sets, cDNA sequences were downloaded from Ensembl (5) and Ensembl genomes (6) (downloaded on 2018-11-15), and subsequently filtered using UniProt's ID mapping (7) and Ensembl REST API cross-reference functionality to retain sequences matching with proteins from the GO annotation files exclusively. Each input data set was processed using TRAPID 2.0, selecting either PLAZA 4.5 dicots or eggNOG 4.5 as reference database. Taxonomic classification and non-coding RNA identification were disabled and all remaining initial processing parameters set to their default values. For PLAZA 4.5 dicots, the impact of the GF transfer cutoff, the minimum frequency among the members of a GF required for a functional annotation label to be assigned to a transcript set to 0.5 in TRAPID 2.0, was assessed. In total, 11 GF transfer cutoff were tested, ranging from 0 (i.e. the functional annotation labels associated to any member of the GF are transferred to the transcript) to 1 (i.e. only functional annotation labels that are associated to all the members of a GF are transferred to the transcript).

For each cDNA sequence, functional annotation performance was evaluated by comparing the set of predicted GO terms to the 'gold standard' GO terms, here defined as the GO terms associated to the corresponding protein with experimental, IC (Inferred by Curator), or TAS (Traceable Author Statement) GO evidence codes. Sensitivity, precision, and F_1 score were computed as described in (8), for each GO aspect. To produce single evaluation values per organism, GO aspect, and experimental setting, the metrics computed for individual cDNA sequences were averaged across all the sequences of an organism (ignoring those for which evaluation metrics were impossible to get). In addition to the functional annotation performance metrics, the annotation coverage, corresponding to the fraction of sequences for which GO terms were predicted, was also computed.

To prevent biasing the functional annotation evaluation metrics with frequent and broad GO terms not carrying a lot of information, 86 GO terms were filtered out and ignored in the benchmark experiments. The set of filtered GO terms consists of the three root GO terms ('cellular_component', 'molecular_function', 'biological_process'), and 83 GO terms that were associated to at least 20% of the proteins of any of the considered organisms in the 'true' GO annotations.

Supplementary Note S3. Eukaryotic gene space completeness comparison

TRAPID 2.0's 'core GF' gene space completeness module and BUSCO (9) were used to estimate the eukaryotic gene space completeness of 26 microbial eukaryote transcriptomes from the MMETSP. For each sample, the core eukaryotic completeness score was computed using 1,116 'core' eggNOG orthologous groups conserved in at least 90% of the eukaryote organisms (see Material and Methods), after initial processing was performed with eggNOG 4.5 as a reference database and default parameters. The completeness score was computed in two settings: using all transcripts of the transcriptome, and excluding those flagged as 'partial' (fragmented). Each sample was additionally processed using BUSCO 4.0.2, executed in transcriptome mode with the 'eukaryota_odb10' lineage dataset (255 quasi single-copy orthologous groups defined across 70 organisms) and default parameters. The resulting eukaryotic gene space completeness scores were subsequently extracted and compared.

As illustrated in Supplementary Figure S5A, the eukaryotic gene space completeness scores computed with TRAPID's core GF procedure and BUSCO are moderately correlated (Pearson correlation coefficient of 0.679). Core GF scores are overall higher, and several samples show stark differences, such as MMETSP0738 (*Thalassiosira*) with a core GF score of 84.1% and a much lower BUSCO score of 13.3%. The score differences are likely due to two main factors: (i) different underlying data sets used to model the expected gene content (in terms of taxonomic coverage, number of genes and filtering on copy number), and (ii) methodological differences. The fact that BUSCO excludes 'fragmented' gene models from the final completeness score, which can represent a substantial part of a *de novo* transcriptome, seems to explain most of the observed differences. When discarding transcripts flagged as 'partial' by TRAPID 2.0, the completeness scores are strongly correlated (Pearson correlation coefficient of 0.904, see Supplementary Figure S5B).

SUPPLEMENTARY FIGURES

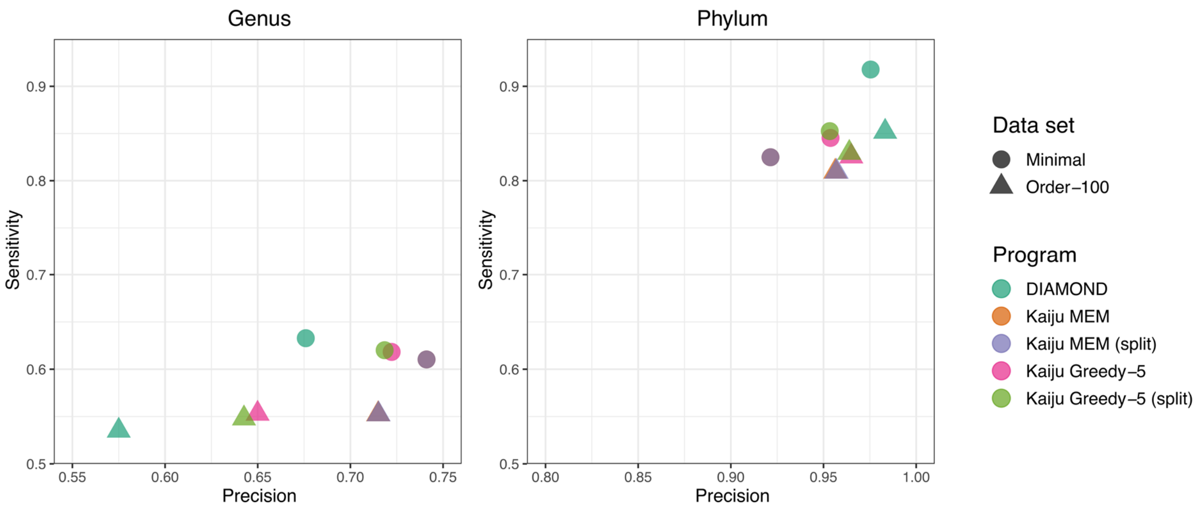

Supplementary Figure S1. Taxonomic classification evaluation, reporting genus- and phylum-level sensitivity and precision. For all evaluated programs, sensitivity and precision values were averaged over replicates of each type of evaluation data set.

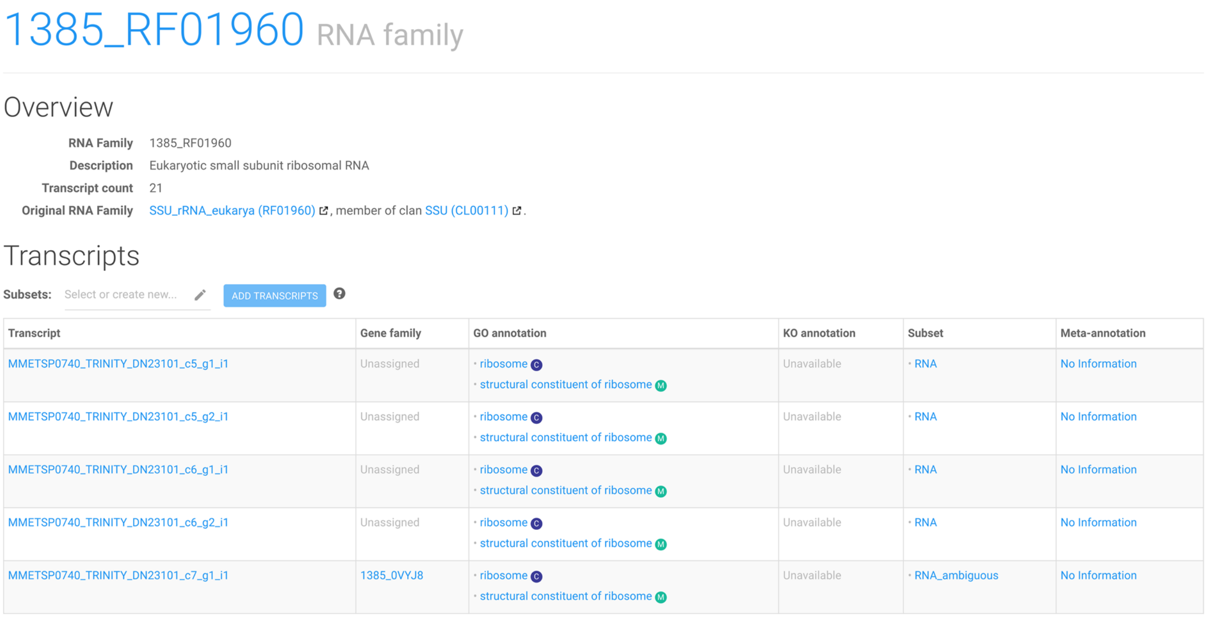

Supplementary Figure S2. RNA family page screenshot. Twenty-one transcripts, listed in the table, were assigned to the 'RF01960' RNA family ('SSU_rRNA_eukarya'). Links to Rfam pages for this family and its encompassing clan are available, and further investigation of individual transcripts is possible. The form on top of the table enables the addition of transcripts to an existing or new subset. GO terms associated to the RNA family were transferred to the transcript sequences ('GO annotation' column). Only five transcripts of the table are shown to improve legibility. These results were generated using MMETSP0740 (*Thalassiosira minuscula* CCMP1093) with eggNOG 4.5 as a reference database and default initial processing parameters.

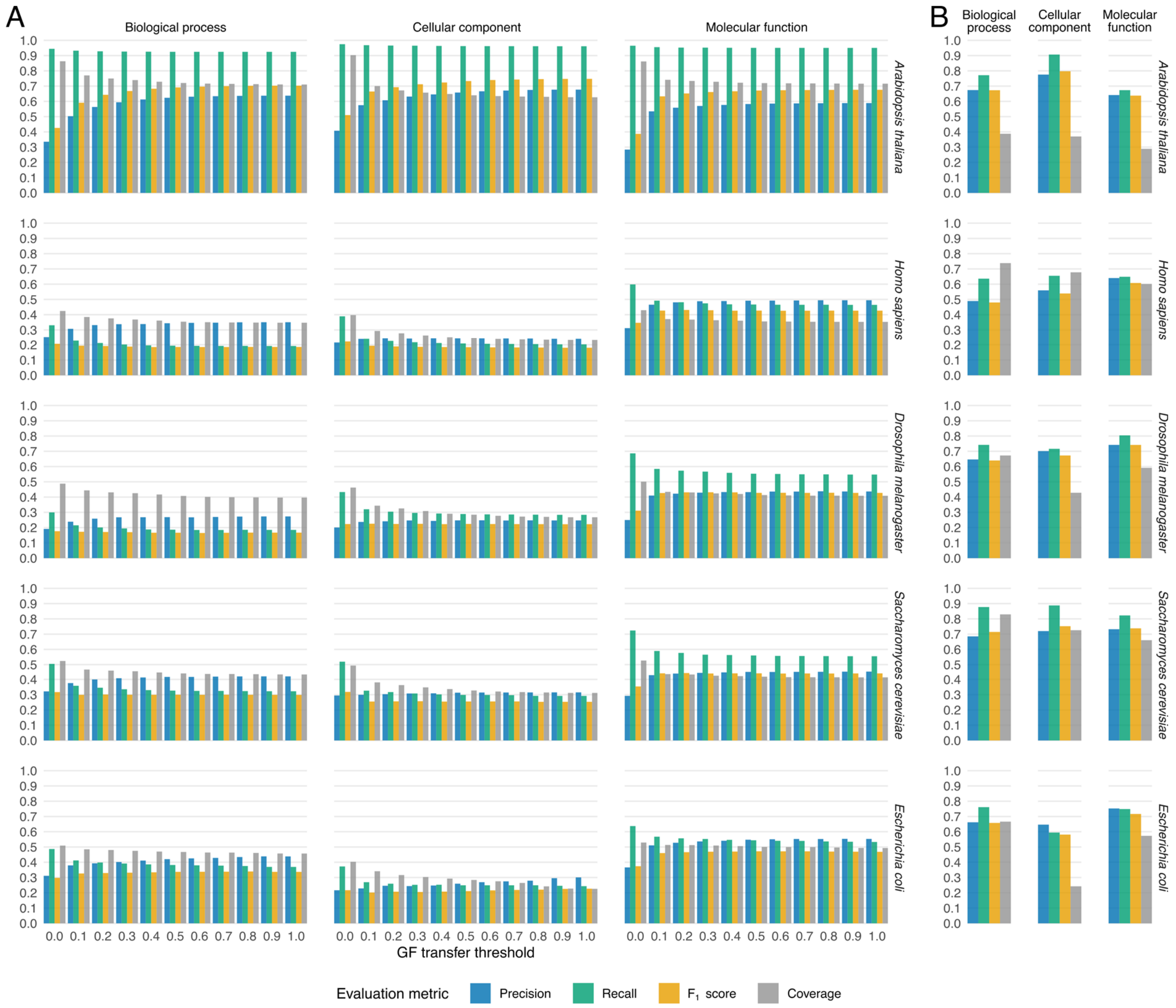

Supplementary Figure S3. TRAPID 2.0 GO annotation performance evaluation. GO annotation evaluation metrics yielded when selecting PLAZA 4.5 dicots as a reference database (**A**), testing varying gene family transfer thresholds, or eggNOG 4.5 (**B**). Each row corresponds to one of the five considered model organisms, and each column to a GO aspect.

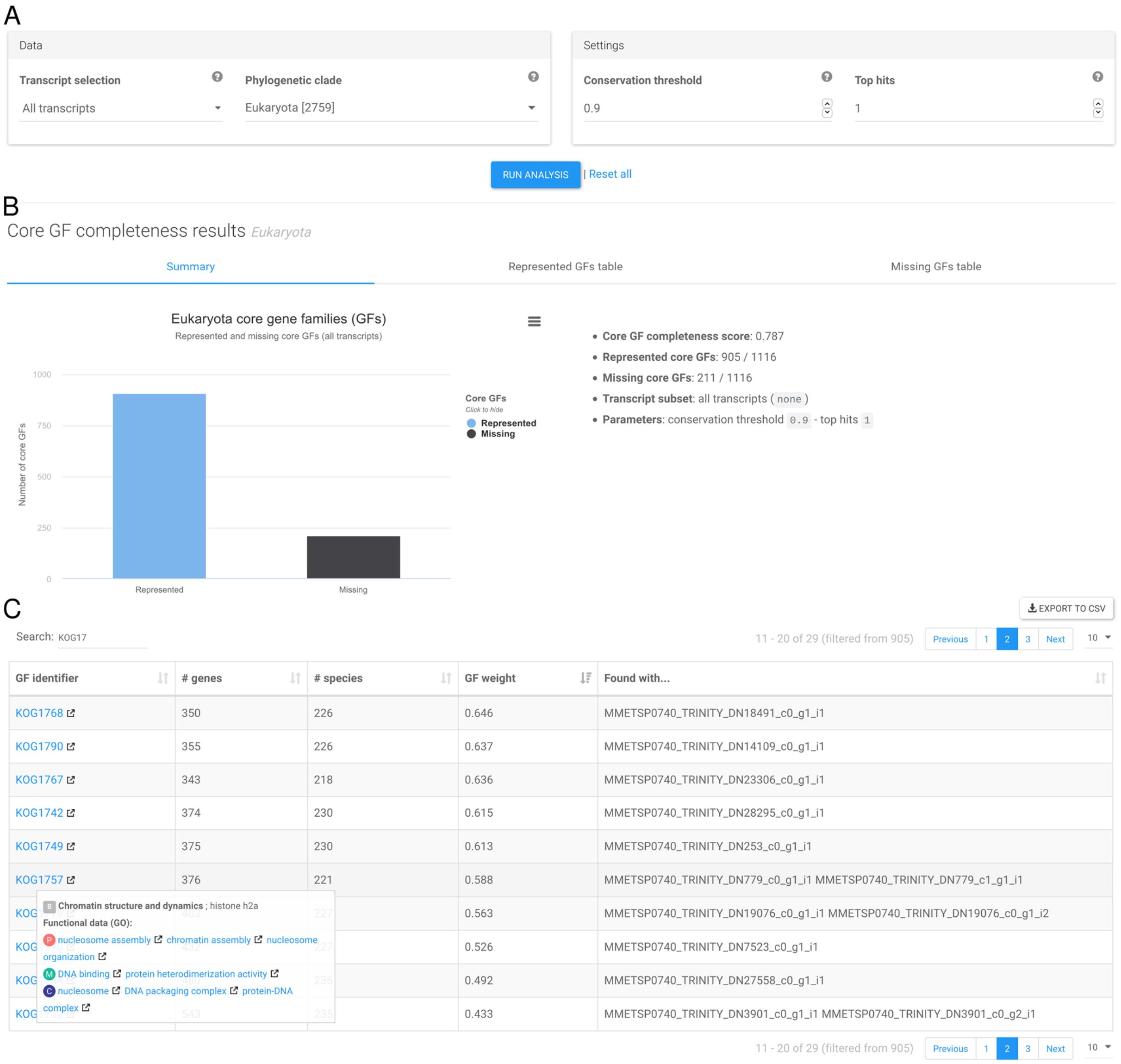

Supplementary Figure S4. Core gene family completeness analysis module screenshot. (**A**) Job submission panel. Any phylogenetic clade represented in the selected reference database can be used for the analysis. The species conservation threshold, used to define core gene families, is also adjustable to fit variable stringency requirements. (**B**) Analysis results panel. Results are summarized as a bar chart depicting the number of represented and missing core gene families, the completeness score and additional analysis metrics. (**C**) Represented core gene families table. Represented or missing core gene families and their associated functional data can be further investigated using the dedicated tables. These results were generated using MMETSP0740 (*Thalassiosira minuscula* CCMP1093) with eggNOG 4.5 as a reference database.

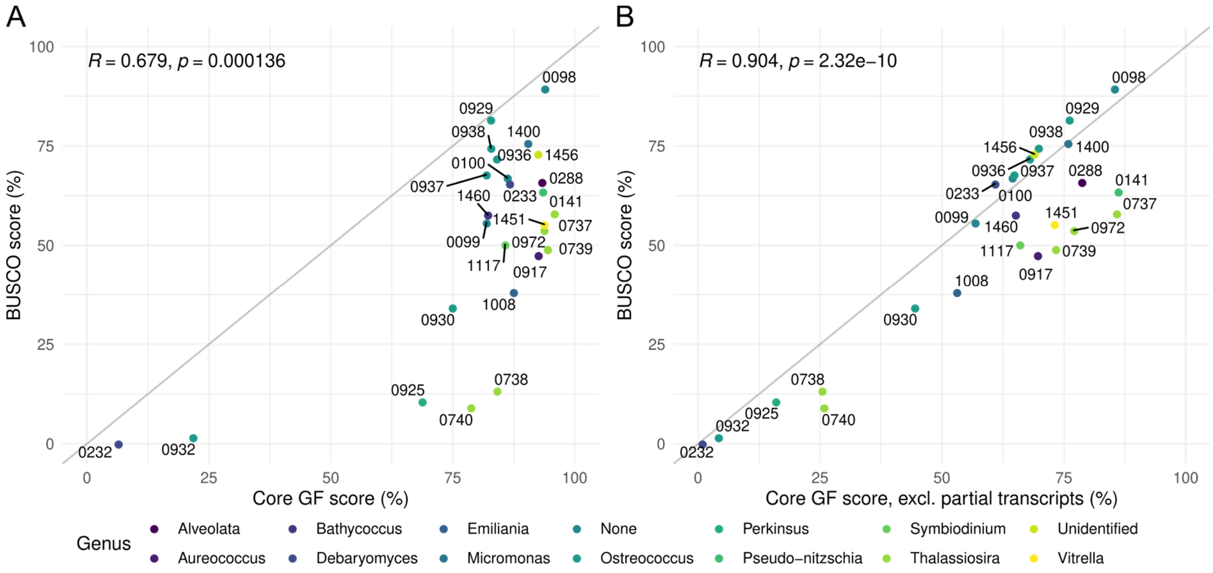

Supplementary Figure S5. Comparison of core GF completeness and BUSCO scores for 26 MMETSP transcriptomes. (**A**) Scatterplot of core eukaryotic completeness score (all transcripts, x-axis) and BUSCO score (y-axis). (**B**) Scatterplot of core eukaryotic completeness score (excluding 'partial' transcripts, x-axis) and BUSCO score (y-axis). Samples are colored by genus, as retrieved from the MMETSP metadata, and complemented by their numerical identifier.

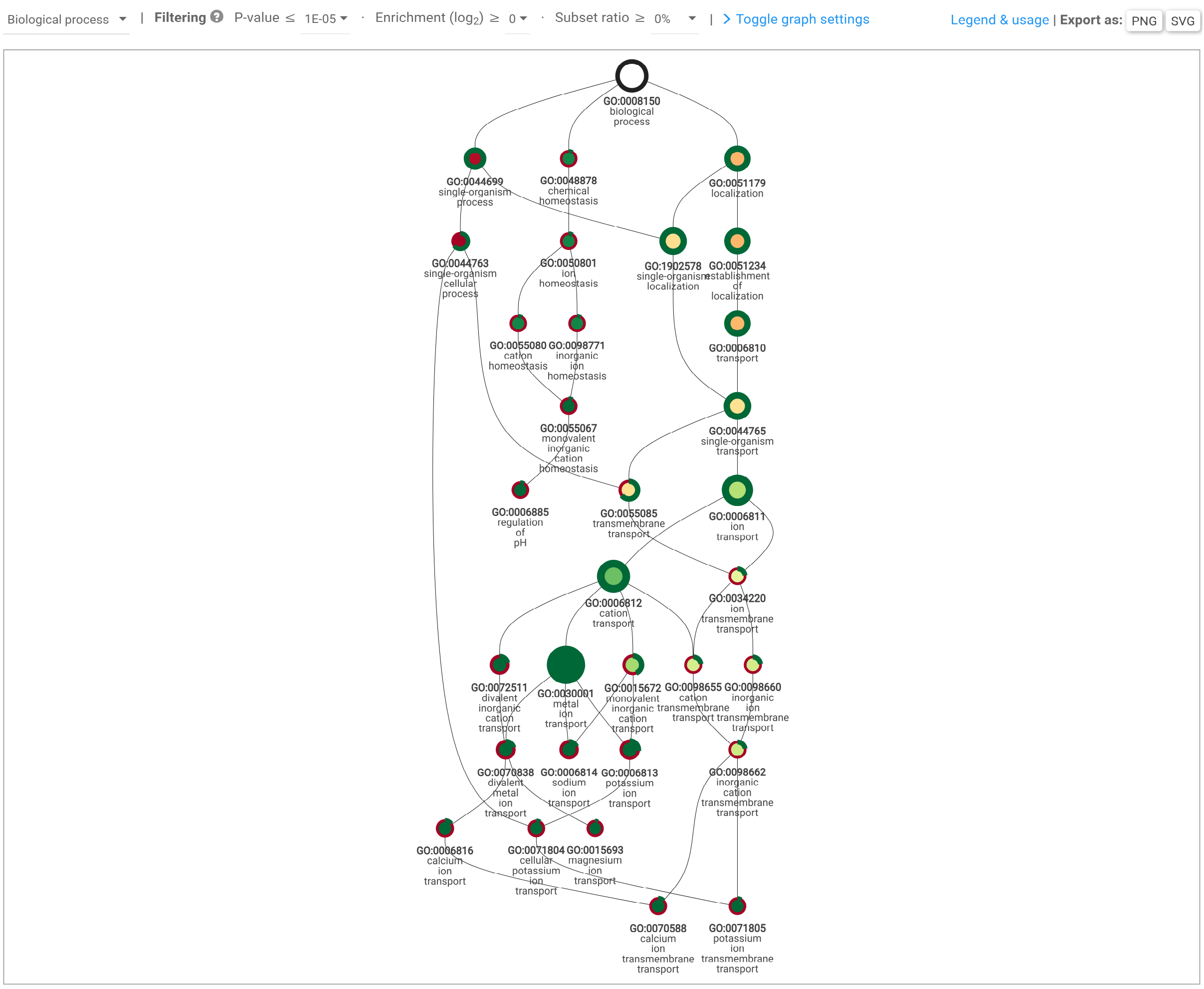

Supplementary Figure S6. Interactive subgraph of enriched GO terms for a subset of 48 *Ostreococcus mediterraneus* metal ion transport transcripts. The nodes represent enriched GO terms and are arranged hierarchically, with ancestral terms (more general) displayed on top and descendant terms (more specialized) at the bottom. The node size is proportional to the GO term's enrichment *q*-value, with a larger size indicating a lower *q*-value (i.e., a more significant enrichment). The node color corresponds to the GO term's enrichment fold, ranging from red (lowest) to green (highest). The node outline depends on the number of transcripts annotated with the GO term in the subset: the green part represents the fraction of transcripts in which it is present and the red part the fraction in which it is absent. The control panel displayed on top of the graph enables the user to choose the GO aspect to visualize (here biological process), filter nodes based on minimum or maximum values of enrichment metrics, and adjust the appearance of the graph. Clicking any GO identifier on the graph opens the dedicated GO term page. Hovering over a node displays a tooltip detailing the corresponding enrichment metrics, and right-clicking it shows a menu with node selection and hiding options. These results were generated using MMETSP0936 (*Ostreococcus mediterraneus* clade-D-RCC2573) with pico-PLAZA 3.0 as a reference database and default initial processing parameters.

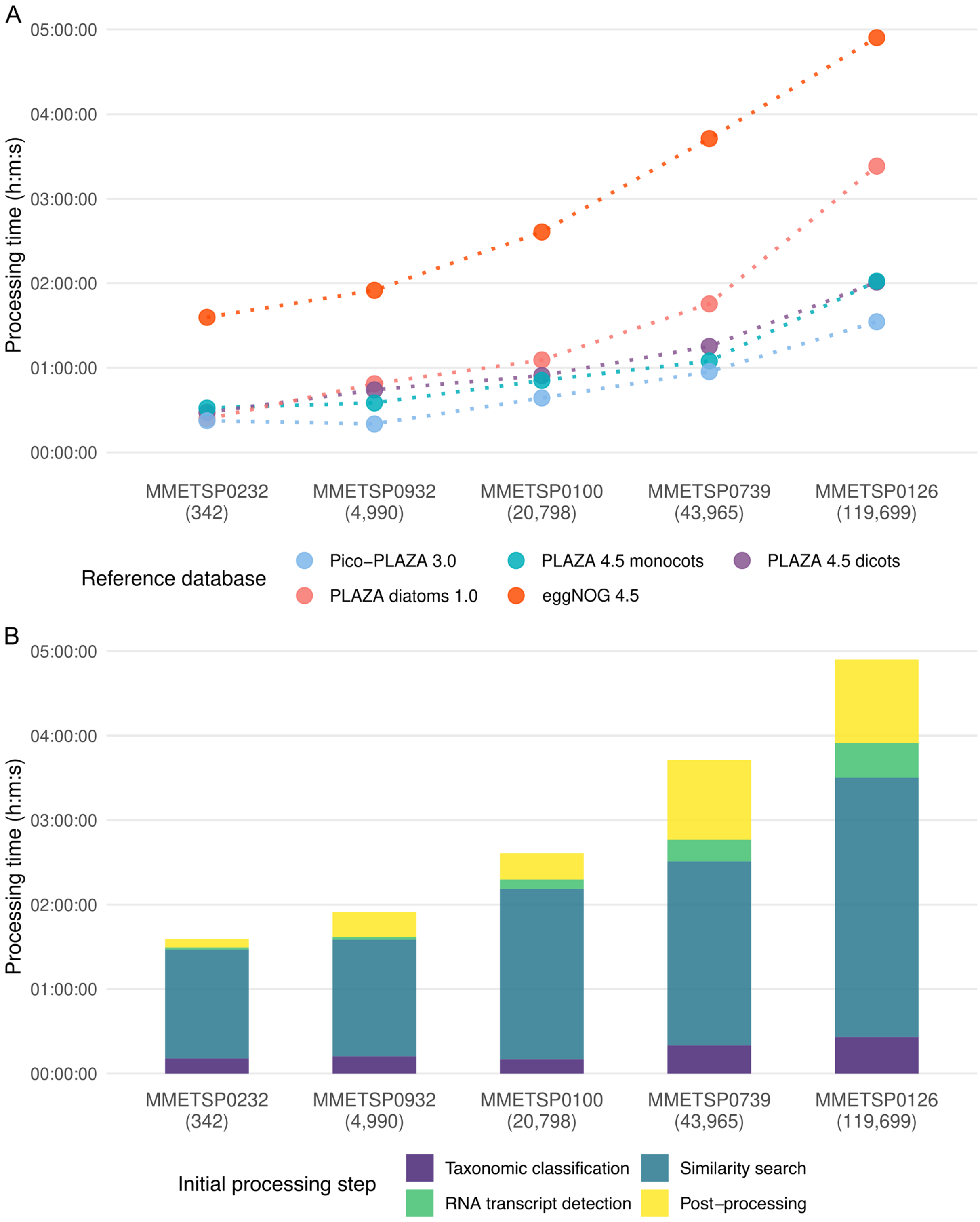

Supplementary Figure S7. TRAPID 2.0 initial processing execution time for five MMETSP transcriptomes. (**A**) Total initial processing execution time for all tested transcriptomes and available reference databases. (**B**) Execution time per initial processing step, for all tested transcriptomes and using eggNOG 4.5 as reference database. The transcript count of each transcriptome is indicated between brackets. All initial processing parameters were set to their default values. The reported execution times are average values obtained over four runs.

SUPPLEMENTARY TABLES

Supplementary Table S1. Sample name, transcript count, and taxonomy of processed MMETSP transcriptomes. Sample taxonomy information was retrieved from the MMETSP metadata.

Sample name	#transcripts	Phylum	Genus	Species	Strain
**MMETSP0018**	19,640	Ciliophora	Uronema	sp.	Bbcil
**MMETSP0098**	45,351	Unknown	None	None	NY0313808BC1
**MMETSP0099**	17,555	Unknown	None	None	NY0313808BC1
**MMETSP0100**	20,798	Unknown	None	None	NY0313808BC1
**MMETSP0123**	38,054	Ciliophora	Favella	ehrenbergii	Fehren 1
**MMETSP0125**	41,818	Ciliophora	Aristerostoma	sp.	ATCC 50986
**MMETSP0126**	119,699	Ciliophora	Strombidinopsis	acuminatum	SPMC142
**MMETSP0127**	83,965	Ciliophora	Platyophrya	macrostoma	WH
**MMETSP0141**	28,457	Bacillariophyta	Pseudo-nitzschia	australis	10249 10 AB
**MMETSP0208**	42,764	Ciliophora	Strombidium	inclinatum	S3
**MMETSP0211**	44,971	Ciliophora	Pseudokeronopsis	sp.	OXSARD2
**MMETSP0216**	65,706	Ciliophora	Protocruzia	adherens	Boccale
**MMETSP0232**	342	Ascomycota	Debaryomyces	hansenii	J26
**MMETSP0233**	9,596	Ascomycota	Debaryomyces	hansenii	J26
**MMETSP0288**	45,732	Apicomplexa	Alveolata	sp.	CCMP3155
**MMETSP0434**	48,003	Ciliophora	Favella	taraikaensis	Fe Narragansett Bay
**MMETSP0436**	33,913	Ciliophora	Favella	taraikaensis	Fe Narragansett Bay
**MMETSP0449**	19,501	Ciliophora	Strombidium	rassoulzadegani	ras09
**MMETSP0463**	43,507	Ciliophora	Strombidinopsis	sp.	SopsisLIS2011
**MMETSP0472**	135,916	Ciliophora	Tiarina	fusus	LIS
**MMETSP0737**	47,167	Bacillariophyta	Thalassiosira	miniscula	CCMP1093
**MMETSP0738**	34,790	Bacillariophyta	Thalassiosira	miniscula	CCMP1093
**MMETSP0739**	43,965	Bacillariophyta	Thalassiosira	miniscula	CCMP1093
**MMETSP0740**	36,324	Bacillariophyta	Thalassiosira	miniscula	CCMP1093
**MMETSP0917**	32,246	Ochrophyta	Aureococcus	anophagefferens	CCMP1850
**MMETSP0925**	29,341	Perkinsozoa	Perkinsus	chesapeaki	ATCC PRA-65
**MMETSP0929**	9,005	Chlorophyta	Ostreococcus	mediterraneus	clade-D-RCC2572
**MMETSP0930**	9,482	Chlorophyta	Ostreococcus	mediterraneus	clade-D-RCC1621
**MMETSP0932**	4,990	Chlorophyta	Ostreococcus	mediterraneus	clade-D-RCC2596
**MMETSP0936**	9,258	Chlorophyta	Ostreococcus	mediterraneus	clade-D-RCC2573
**MMETSP0937**	9,289	Chlorophyta	Ostreococcus	mediterraneus	clade-D-RCC2593
**MMETSP0938**	9,193	Chlorophyta	Ostreococcus	mediterraneus	clade-D-RCC1107
**MMETSP0972**	37,371	Bacillariophyta	Thalassiosira	oceanica	CCMP1005
**MMETSP1008**	35,540	Haptophyta	Emiliania	huxleyi	374
**MMETSP1018**	17,679	Ciliophora	Anophryoides	haemophila	AH6
**MMETSP1019**	21,468	Ciliophora	Anophryoides	haemophila	AH6
**MMETSP1117**	64,980	Dinophyta	Symbiodinium	sp.	CCMP2430
**MMETSP1396**	49,169	Ciliophora	Pseudokeronopsis	sp.	Brazil
**MMETSP1400**	14,968	Chlorophyta	Micromonas	sp.	RCC451
**MMETSP1451**	54,204	Alveolata	Vitrella	brassicaformis	CCMP3346
**MMETSP1456**	12,219	Unknown	Unidentified	sp.	RCC1871
**MMETSP1460**	11,309	Chlorophyta	Bathycoccus	prasinos	RCC716

Supplementary Table S2. Overview of TRAPID 2.0 reference databases. Gene counts only include protein-coding genes. Highlighted rows illustrate the taxonomic focus and complementarity of the available reference databases.

Reference database	Gene family construction	Functional annotation	Taxonomic focus	Clade	#Species	#Genes
PLAZA 4.5 dicots[1]	Tribe-MCL, integrative orthologs	Gene Ontology terms, InterPro domains	Dicot plants	Viridiplantae	55	1,833,029
				Magnoliophyta	49	1,664,055
				Eudicotyledons	46	1,555,522
PLAZA 4.5 monocots[2]	Tribe-MCL, integrative orthologs	Gene Ontology terms, InterPro domains	Monocot plants	Viridiplantae	39	1,497,121
				Magnoliophyta	33	1,317,149
				Liliopsida	28	1,158,627
pico-PLAZA 3.0[3]	Tribe-MCL, integrative orthologs	Gene Ontology terms, InterPro domains	Microbial photosynthetic eukaryotes	Eukaryota	39	572,836
				Chlorophyta	16	150,663
				Opisthokonta	6	82,089
				Rhodophyta	3	21,995
				SAR	8	138,838
				Viridiplantae	20	278,148
PLAZA Diatoms 1.0[4]	Tribe-MCL, integrative orthologs	Gene Ontology terms, InterPro domains	Diatoms	Eukaryota	26	503,959
				Bacilliarophyta	10	217,184
				Opisthokonta	5	68,734
				Rhodophyta	3	22,144
				SAR	14	296,444
				Viridiplantae	3	78,083
eggNOG 4.5[5]	eggNOG	Gene Ontology terms, KEGG Orthology groups	Archaea, Bacteria, Eukaryotes	Cellular organisms	2,031	9,646,196
				Archaea	115	256,635
				Bacteria	1,678	5,559,847
				Eukaryota	238	3,829,714
				Apicomplexa	13	67,347
				Euglenozoa	7	69,150
				Fungi	85	779,911
				Metazoa	89	1,714,272
				Oomycota	6	95,774
				Viridiplantae	23	810,696

[1] https://bioinformatics.psb.ugent.be/plaza/versions/plaza_v4_5_dicots
[2] https://bioinformatics.psb.ugent.be/plaza/versions/plaza_v4_5_monocots
[3] https://bioinformatics.psb.ugent.be/plaza/versions/plaza_pico_03
[4] https://bioinformatics.psb.ugent.be/plaza/versions/plaza_diatoms_01
[5] http://eggnog45.embl.de

Supplementary Table S3. Read filtering and mapping information for three Antarctic Peninsula phytoplankton community metatranscriptomes from the Bransfield Strait (BFS), Wilkins sea ice (WKI) and Weddell Sea (WDS) samples. Raw reads from the three samples were filtered and trimmed, and used for metatranscriptome assembly and expression quantification. The values reported in the '# Suppl. Alignments' and the '#Unmapped in unused' columns correspond to the number of mapped reads having supplementary alignments, and the number of unmapped reads that are in data that is unused in the assembly, respectively.

Source	SRA accession	#Raw reads	#Quality-filtered reads (% raw reads)	#Mapped (% retained reads)	#Suppl. alignments (% retained reads)	#Unmapped (% retained reads)	#Unmapped in unused (% retained reads)
BFS	SRR1606327	114,323	108,291 (94.72)	88,426 (81.66)	174 (0.16)	19,865 (18.34)	18,100 (16.71)
WDS	SRR1606329	253,631	239,196 (94.31)	185,275 (77.46)	375 (0.16)	53,921 (22.54)	48,809 (20.41)
WKI	SRR1606328	191,057	179,040 (93.71)	137,764 (76.95)	183 (0.10)	41,276 (23.05)	37,333 (20.85)
Total	-	559,011	526,527 (94.19)	411,465 (78.15)	732 (0.14)	115,062 (21.85)	104,242 (19.80)

Supplementary Table S4. Overview of all TRAPID 2.0 processing steps and their purpose. Processing steps are organized by processing phase, initial processing or exploratory, and chronological execution order when possible. Indentation indicates hierarchical relationships between processing steps.

Processing phase	Processing step	Purpose
**Initial processing phase**		Global transcriptome characterization
	Taxonomic classification	Potential contaminant detection; characterization of communities
	RNA transcripts detection	Non-coding RNA identification and annotation
	Sequence similarity search	Identification of homologous sequences within available reference databases
	Sequence similarity search post-processing	Characterization of protein-coding transcripts
	– Gene Family assignment	Clustering of transcripts with their homologs
	– Functional annotation transfer	Functional annotation of transcript sequences
	– Meta-annotation inference	Estimation of the full-length status of transcripts
	– Frame detection and ORF finding	Putative frameshift detection; coding sequence detection and translation (supporting non-canonical genetic codes)
**Exploratory phase**		Refined comparative and functional analyses; examination of selected data types
	Experiment statistics	Exploration of annotation metrics and sequence length distribution
	Global protein alignment and phylogenetic tree construction	Analysis of transcripts and their homologs in a gene-family and evolutionary context; identification of orthology and paralogy relationships
	Core GF completeness analysis	Gene space completeness assessment and analysis at multiple phylogenetic levels
	Subset functional enrichment analysis	Identification and analysis of functional biases; discovery and exploration of significantly over-represented biological functions
	Subset functional annotation comparison	Exploration of the biological function of transcript subsets; comparison of functional biases

Supplementary Table S5. 'Minimal' taxonomic classification evaluation dataset composition and sampling proportions. Sequence counts indicate the number of available RNA sequences from each sampled clade in RefSeq (release 84).

Clade (NCBI taxon identifier)	Rank	#sequences	Fraction
Chlorophyta (3041)	Phylum	115,801	0.20
Streptophyta (35493)	Phylum	4,190,204	0.20
Bacteria (2)	Superkingdom	21,190	0.15
Chordata (7711)	Phylum	9,494,012	0.05
Basidiomycota (5204)	Phylum	486,296	0.05
Arthropoda (6656)	Phylum	2,524,444	0.05
Ascomycota (4890)	Phylum	1,685,068	0.05
Mollusca (6447)	Phylum	218,941	0.05
Apicomplexa (5794)	Phylum	186,627	0.05
Nematoda (6231)	Phylum	166,442	0.05
Cnidaria (6073)	Phylum	154,324	0.05
Archaea (2157)	Superkingdom	1,162	0.05

Supplementary Table S6. TRAPID 2.0 experiment statistics summary for the global Antarctic Peninsula phytoplankton community metatranscriptome.

Metric	Value
**Transcript information**	
#Transcripts	143,308
Average sequence length	308
Average ORF length	245.9
#Partial (% total)	44,248 (30.9%)
**Taxonomic classification information**	
#Classified (% total)	77,105 (53.8%)
#Unclassified (% total)	66,203 (46.2%)
**Similarity search information (top 5)**	
Fragilariopsis cylindrus (% total)	16,908 (26.57%)
Thalassiosira pseudonana (% total)	13,796 (21.68%)
Phaeodactylum tricornutum (% total)	8,919 (14.01%)
Drosophila melanogaster (% total)	2,651 (4.17%)
Emiliania huxleyi (% total)	2,132 (3.35%)
Total hits (% total)	63,641 (44.41%)
**Gene family information**	
#Gene families	8,931
#Transcripts in GF (% total)	63,641 (44.4%)
Largest GF	HOM03P000062 (602 transcripts)
#Single copy	3,409
**RNA family information**	
#RNA families	13
#Transcripts in RF (% total)	4,536 (3.2%)
Largest RF	RF02543 (1,498 transcripts)
**Functional annotation information**	
#GO terms	12,965
#Transcripts with GO (% total)	65,831 (45.9%)
#InterPro domains	7,052
#Transcripts with Protein Domain (% total)	63,641 (44.4%)

Supplementary Table S7. Enriched InterPro domains in diatom-assigned transcripts expressed in the Bransfield Strait (BFS) sample, compared with the whole Antarctic Peninsula phytoplankton community metatranscriptome. Enrichment fold values are expressed in log_2 scale and the subset ratio indicates the percentage of transcripts annotated with the enriched functional annotation label among all the annotated transcripts of the subset. Maximum enrichment *q*-value threshold is 0.01.

Identifier	*q*-value	Enrichment fold	Subset ratio	Description
IPR011989	6.568E-10	0.733	2.875	Armadillo-like helical
IPR013537	1.595E-09	2.117	0.390	Acetyl-CoA carboxylase, central domain
IPR016024	2.301E-09	0.610	3.742	Armadillo-type fold
IPR023329	4.040E-08	0.633	3.005	Chlorophyll a/b binding protein domain
IPR022796	5.224E-08	0.632	2.947	Chlorophyll A-B binding protein
IPR011054	8.971E-08	1.372	0.693	Rudiment single hybrid motif
IPR005481	3.978E-07	1.608	0.477	Biotin carboxylase-like, N-terminal domain
IPR005482	3.978E-07	1.608	0.477	Biotin carboxylase, C-terminal
IPR011764	3.978E-07	1.608	0.477	Biotin carboxylation domain
IPR001882	4.484E-07	1.591	0.477	Biotin-binding site
IPR000022	1.302E-06	1.717	0.390	Carboxyl transferase
IPR011763	1.761E-06	1.695	0.390	Acetyl-coenzyme A carboxyltransferase, C-terminal
IPR011762	2.376E-06	1.674	0.390	Acetyl-coenzyme A carboxyltransferase, N-terminal
IPR005479	2.628E-06	1.235	0.665	Carbamoyl-phosphate synthetase large subunit-like, ATP-binding domain
IPR016185	4.922E-06	1.103	0.780	Pre-ATP-grasp domain
IPR029045	7.531E-05	1.021	0.737	ClpP/crotonase-like domain
IPR000089	1.067E-04	1.113	0.607	Biotin/lipoyl attachment
IPR009080	1.119E-04	1.072	0.650	Aminoacyl-tRNA synthetase, class 1a, anticodon-binding
IPR011053	1.215E-04	1.104	0.607	Single hybrid motif
IPR002300	1.565E-04	1.313	0.433	Aminoacyl-tRNA synthetase, class Ia
IPR014729	1.805E-04	0.642	1.560	Rossmann-like alpha/beta/alpha sandwich fold
IPR009008	1.842E-04	1.325	0.419	Valyl/Leucyl/Isoleucyl-tRNA synthetase, editing domain
IPR013155	2.144E-04	1.310	0.419	Methionyl/Valyl/Leucyl/Isoleucyl-tRNA synthetase, anticodon-binding
IPR013041	6.156E-04	1.443	0.318	Coatomer/clathrin adaptor appendage, Ig-like subdomain
IPR011990	6.323E-04	0.482	2.355	Tetratricopeptide-like helical domain
IPR005475	8.717E-04	0.998	0.592	Transketolase-like, pyrimidine-binding domain
IPR011761	8.945E-04	0.812	0.852	ATP-grasp fold
IPR001412	9.054E-04	0.944	0.650	Aminoacyl-tRNA synthetase, class I, conserved site
IPR013815	1.208E-03	0.836	0.780	ATP-grasp fold, subdomain 1
IPR002553	1.269E-03	1.222	0.390	Clathrin/coatomer adaptor, adaptin-like, N-terminal
IPR002885	1.281E-03	0.955	0.592	Pentatricopeptide repeat
IPR000185	1.367E-03	1.321	0.332	Protein translocase subunit SecA
IPR011115	1.367E-03	1.321	0.332	SecA DEAD-like, N-terminal
IPR011116	1.367E-03	1.321	0.332	SecA Wing/Scaffold
IPR011130	1.367E-03	1.321	0.332	SecA preprotein, cross-linking domain
IPR014018	1.367E-03	1.321	0.332	SecA motor DEAD
IPR020937	1.367E-03	1.321	0.332	SecA conserved site
IPR001478	1.395E-03	0.860	0.708	PDZ domain
IPR017932	1.425E-03	1.016	0.535	Glutamine amidotransferase type 2 domain
IPR003440	1.510E-03	2.080	0.144	Glycosyl transferase, family 48
IPR026899	1.510E-03	2.080	0.144	1,3-beta-glucan synthase subunit FKS1-like, domain-1
IPR007066	1.593E-03	1.076	0.462	RNA polymerase Rpb1, domain 3
IPR007081	1.593E-03	1.076	0.462	RNA polymerase Rpb1, domain 5
IPR007083	1.593E-03	1.076	0.462	RNA polymerase Rpb1, domain 4
IPR029061	2.251E-03	0.784	0.780	Thiamin diphosphate-binding fold
IPR019734	2.282E-03	0.604	1.257	Tetratricopeptide repeat
IPR009014	4.751E-03	0.928	0.520	Transketolase, C-terminal/Pyruvate-ferredoxin oxidoreductase, domain II
IPR005467	6.044E-03	1.199	0.318	Histidine kinase domain
IPR005113	6.123E-03	1.487	0.217	uDENN domain
IPR001194	6.430E-03	1.421	0.231	DENN domain

IPR001296	6.468E-03	1.765	0.159	Glycosyl transferase, family 1
IPR000403	6.510E-03	1.132	0.347	Phosphatidylinositol 3-/4-kinase, catalytic domain
IPR010788	7.233E-03	2.100	0.116	VDE lipocalin domain
IPR001108	9.669E-03	2.421	0.087	Peptidase A22A, presenilin

Supplementary Table S8. Enriched InterPro domains in diatom-assigned transcripts expressed in the Weddell Sea (WDS) sample, compared with the whole Antarctic Peninsula phytoplankton community metatranscriptome. Enrichment fold values are expressed in $\log_2$ scale and the subset ratio indicates the percentage of transcripts annotated with the enriched functional annotation label among all the annotated transcripts of the subset. Maximum enrichment *q*-value threshold is 0.01.

Identifier	*q*-value	Enrich-ment fold	Subset ratio	Description
IPR023329	9.883E-60	1.278	4.699	Chlorophyll a/b binding protein domain
IPR022796	9.554E-58	1.269	4.583	Chlorophyll A-B binding protein
IPR003959	1.407E-11	0.836	2.151	ATPase, AAA-type, core
IPR005804	1.041E-10	1.347	0.832	Fatty acid desaturase domain
IPR002146	2.015E-09	2.118	0.320	ATPase, F0 complex, subunit B/B', bacterial/chloroplast
IPR000185	2.438E-07	1.623	0.410	Protein translocase subunit SecA
IPR011115	2.438E-07	1.623	0.410	SecA DEAD-like, N-terminal
IPR011116	2.438E-07	1.623	0.410	SecA Wing/Scaffold
IPR011130	2.438E-07	1.623	0.410	SecA preprotein, cross-linking domain
IPR014018	2.438E-07	1.623	0.410	SecA motor DEAD
IPR020937	2.438E-07	1.623	0.410	SecA conserved site
IPR006424	4.081E-07	1.056	0.845	Glyceraldehyde-3-phosphate dehydrogenase, type I
IPR020828	4.081E-07	1.056	0.845	Glyceraldehyde 3-phosphate dehydrogenase, NAD(P) binding domain
IPR020829	4.081E-07	1.056	0.845	Glyceraldehyde 3-phosphate dehydrogenase, catalytic domain
IPR020830	4.081E-07	1.056	0.845	Glyceraldehyde 3-phosphate dehydrogenase, active site
IPR020831	4.081E-07	1.056	0.845	Glyceraldehyde/Erythrose phosphate dehydrogenase family
IPR000642	1.002E-06	1.319	0.525	Peptidase M41
IPR000685	1.031E-06	1.865	0.282	Ribulose bisphosphate carboxylase, large subunit, C-terminal
IPR017443	1.031E-06	1.865	0.282	Ribulose bisphosphate carboxylase, large subunit, ferrodoxin-like N-terminal
IPR020878	1.031E-06	1.865	0.282	Ribulose bisphosphate carboxylase, large chain, active site
IPR005936	1.378E-06	1.321	0.512	Peptidase, FtsH
IPR000932	1.700E-06	1.258	0.551	Photosystem antenna protein-like
IPR003593	6.505E-06	0.388	4.391	AAA+ ATPase domain
IPR015701	6.701E-06	1.578	0.333	Ferredoxin--NADP reductase
IPR002938	1.788E-05	1.247	0.474	FAD-binding domain
IPR027417	1.851E-05	0.210	12.252	P-loop containing nucleoside triphosphate hydrolase
IPR006082	1.932E-05	1.795	0.243	Phosphoribulokinase
IPR012171	3.614E-05	1.466	0.333	Fatty acid desaturase
IPR005478	7.070E-05	1.313	0.384	Transketolase, bacterial-like
IPR009518	7.504E-05	2.832	0.090	Photosystem II PsbX
IPR005474	8.104E-05	1.233	0.422	Transketolase, N-terminal
IPR020826	8.104E-05	1.233	0.422	Transketolase binding site
IPR001576	1.011E-04	1.358	0.346	Phosphoglycerate kinase
IPR015824	1.011E-04	1.358	0.346	Phosphoglycerate kinase, N-terminal
IPR015901	1.011E-04	1.358	0.346	Phosphoglycerate kinase, C-terminal
IPR015911	1.011E-04	1.358	0.346	Phosphoglycerate kinase, conserved site
IPR010253	2.026E-04	1.588	0.243	Geranylgeranyl reductase, plant/prokaryotic
IPR011774	2.026E-04	1.588	0.243	Geranylgeranyl reductase, plant/cyanobacteria
IPR011777	2.026E-04	1.588	0.243	Geranylgeranyl reductase family
IPR011054	2.635E-04	1.005	0.538	Rudiment single hybrid motif
IPR007066	3.756E-04	1.071	0.461	RNA polymerase Rpb1, domain 3
IPR007081	3.756E-04	1.071	0.461	RNA polymerase Rpb1, domain 5
IPR007083	3.756E-04	1.071	0.461	RNA polymerase Rpb1, domain 4
IPR003960	4.785E-04	0.633	1.165	ATPase, AAA-type, conserved site

IPR011262	4.821E-04	1.417	0.269	DNA-directed RNA polymerase, insert domain
IPR011263	4.821E-04	1.417	0.269	DNA-directed RNA polymerase, RpoA/D/Rpb3-type
IPR009025	4.824E-04	1.385	0.282	DNA-directed RNA polymerase, RBP11-like dimerisation domain
IPR010004	4.894E-04	2.154	0.128	Uncharacterised protein family Ycf66
IPR013185	4.897E-04	1.781	0.179	Translation elongation factor, KOW-like
IPR004540	4.998E-04	1.309	0.307	Translation elongation factor EFG/EF2
IPR020781	5.648E-04	1.832	0.166	ATPase, OSCP/delta subunit, conserved site
IPR000470	6.414E-04	1.562	0.218	CbxX/CfqX, monofunctional
IPR006083	6.435E-04	1.465	0.243	Phosphoribulokinase/uridine kinase
IPR019820	6.594E-04	1.893	0.154	Sec-independent periplasmic protein translocase, conserved site
IPR004176	6.633E-04	1.151	0.371	Clp, N-terminal
IPR018368	6.633E-04	1.151	0.371	ClpA/B, conserved site 1
IPR028299	6.633E-04	1.151	0.371	ClpA/B, conserved site 2
IPR019489	6.633E-04	1.069	0.422	Clp ATPase, C-terminal
IPR007072	7.824E-04	1.584	0.205	Rhamnosyl O-methyltransferase/Cephalosporin hydroxylase
IPR011260	7.824E-04	1.584	0.205	RNA polymerase, alpha subunit, C-terminal
IPR011773	7.824E-04	1.584	0.205	DNA-directed RNA polymerase, alpha subunit
IPR000146	9.772E-04	1.373	0.256	Fructose-1,6-bisphosphatase class 1/Sedoheputulose-1,7-bisphospha-tase
IPR020548	9.772E-04	1.373	0.256	Fructose-1,6-bisphosphatase, active site
IPR028343	9.772E-04	1.373	0.256	Fructose-1,6-bisphosphatase
IPR002033	1.004E-03	1.832	0.154	Sec-independent periplasmic protein translocase TatC
IPR013785	1.041E-03	0.468	1.818	Aldolase-type TIM barrel
IPR000484	1.258E-03	1.116	0.358	Photosynthetic reaction centre, L/M
IPR007265	1.391E-03	2.610	0.077	Conserved oligomeric Golgi complex, subunit 3
IPR000568	1.405E-03	1.417	0.230	ATPase, F0 complex, subunit A
IPR023011	1.405E-03	1.417	0.230	ATPase, F0 complex, subunit A, active site
IPR001280	1.461E-03	1.172	0.320	Photosystem I PsaA/PsaB
IPR020586	1.461E-03	1.172	0.320	Photosystem I PsaA/PsaB, conserved site
IPR001270	1.659E-03	1.012	0.410	ClpA/B family
IPR013005	1.690E-03	1.675	0.166	50S ribosomal protein uL4
IPR004541	1.765E-03	1.387	0.230	Translation elongation factor EFTu/EF1A, bacterial/organelle
IPR016040	1.781E-03	0.289	4.148	NAD(P)-binding domain
IPR000722	1.951E-03	1.169	0.307	RNA polymerase, alpha subunit
IPR006592	1.951E-03	1.169	0.307	RNA polymerase, N-terminal
IPR007080	1.951E-03	1.169	0.307	RNA polymerase Rpb1, domain 1
IPR002325	2.393E-03	1.626	0.166	Cytochrome f
IPR024058	2.393E-03	1.626	0.166	Cytochrome f transmembrane anchor
IPR003672	3.068E-03	1.247	0.256	CobN/magnesium chelatase
IPR011771	3.068E-03	1.247	0.256	Magnesium-chelatase, subunit H
IPR022571	3.068E-03	1.247	0.256	Magnesium chelatase, subunit H, N-terminal
IPR004853	3.131E-03	1.101	0.320	Sugar phosphate transporter domain
IPR001263	3.346E-03	1.454	0.192	Phosphoinositide 3-kinase, accessory (PIK) domain
IPR023222	3.346E-03	1.454	0.192	PsbQ-like domain
IPR024094	3.365E-03	1.578	0.166	Cytochrome f large domain
IPR010251	3.842E-03	2.417	0.077	Magnesium-protoporphyrin IX methyltransferase
IPR010940	3.842E-03	2.417	0.077	Magnesium-protoporphyrin IX methyltransferase, C-terminal
IPR002358	4.366E-03	1.417	0.192	Ribosomal protein L6, conserved site
IPR019906	4.366E-03	1.417	0.192	Ribosomal protein L6, bacterial-type
IPR000711	4.386E-03	1.533	0.166	ATPase, OSCP/delta subunit
IPR005869	4.386E-03	1.533	0.166	Photosystem II CP43 reaction centre protein
IPR026015	4.386E-03	1.533	0.166	F1F0 ATP synthase OSCP/delta subunit, N-terminal domain
IPR005493	4.407E-03	2.180	0.090	Ribonuclease E inhibitor RraA/Dimethylmenaquinone methyltransferase
IPR022147	4.428E-03	1.610	0.154	Glutamine synthetase type III N-terminal
IPR000641	4.466E-03	1.274	0.230	CbxX/CfqX
IPR005706	4.474E-03	1.142	0.282	Ribosomal protein S2, bacteria/mitochondria/plastid
IPR018936	7.415E-03	1.116	0.269	Phosphatidylinositol 3/4-kinase, conserved site
IPR000523	7.765E-03	1.695	0.128	Magnesium chelatase ChlI domain
IPR010136	7.905E-03	2.832	0.051	N-acetyl-gamma-glutamyl-phosphate reductase, type 2
IPR000652	9.706E-03	1.008	0.307	Triosephosphate isomerase
IPR020861	9.706E-03	1.008	0.307	Triosephosphate isomerase, active site
IPR009014	9.807E-03	0.794	0.474	Transketolase, C-terminal/Pyruvate-ferredoxin oxidoreductase, domain II

Supplementary Table S9. Enriched InterPro domains in diatom-assigned transcripts expressed in the Wilkins sea ice (WKI) sample, compared with the whole Antarctic Peninsula phytoplankton community metatranscriptome. Enrichment fold values are expressed in $\log_2$ scale and the subset ratio indicates the percentage of transcripts annotated with the enriched functional annotation label among all the annotated transcripts of the subset. Maximum enrichment *q*-value threshold is 0.01.

Identifier	*q*-value	Enrichment fold	Subset ratio	Description
IPR022796	5.235E-37	0.990	3.778	Chlorophyll A-B binding protein
IPR023329	1.452E-36	0.975	3.810	Chlorophyll a/b binding protein domain
IPR003593	2.960E-08	0.425	4.504	AAA+ ATPase domain
IPR004737	1.271E-07	2.389	0.160	Nitrate transporter
IPR003959	3.236E-07	0.633	1.868	ATPase, AAA-type, core
IPR005936	5.532E-07	1.260	0.491	Peptidase, FtsH
IPR005117	7.081E-07	1.649	0.299	Nitrite/Sulfite reductase ferredoxin-like domain
IPR006066	7.081E-07	1.649	0.299	Nitrite/sulphite reductase iron-sulphur/sirohaem-binding site
IPR006067	7.081E-07	1.649	0.299	Nitrite/sulphite reductase 4Fe-4S domain
IPR000642	7.815E-07	1.223	0.491	Peptidase M41
IPR000185	8.533E-07	1.448	0.363	Protein translocase subunit SecA
IPR011115	8.533E-07	1.448	0.363	SecA DEAD-like, N-terminal
IPR011116	8.533E-07	1.448	0.363	SecA Wing/Scaffold
IPR011130	8.533E-07	1.448	0.363	SecA preprotein, cross-linking domain
IPR014018	8.533E-07	1.448	0.363	SecA motor DEAD
IPR020937	8.533E-07	1.448	0.363	SecA conserved site
IPR017871	1.015E-06	0.603	1.847	ABC transporter, conserved site
IPR003439	1.168E-06	0.576	1.932	ABC transporter-like
IPR021884	4.504E-06	1.136	0.502	Protein of unknown function DUF3494
IPR003960	5.965E-06	0.695	1.217	ATPase, AAA-type, conserved site
IPR006073	6.141E-06	0.888	0.768	GTP binding domain
IPR017926	7.433E-05	0.918	0.598	Glutamine amidotransferase
IPR027417	7.474E-05	0.181	12.008	P-loop containing nucleoside triphosphate hydrolase
IPR007419	1.876E-04	2.122	0.117	BFD-like [2Fe-2S]-binding domain
IPR012744	1.876E-04	2.122	0.117	Nitrite reductase [NAD(P)H] large subunit, NirB
IPR003672	3.972E-04	1.307	0.267	CobN/magnesium chelatase
IPR011771	3.972E-04	1.307	0.267	Magnesium-chelatase, subunit H
IPR022571	3.972E-04	1.307	0.267	Magnesium chelatase, subunit H, N-terminal
IPR029062	5.998E-04	0.790	0.640	Class I glutamine amidotransferase-like
IPR002792	6.225E-04	1.570	0.181	TRAM domain
IPR000183	6.912E-04	1.669	0.160	Ornithine/DAP/Arg decarboxylase
IPR010253	8.916E-04	1.400	0.213	Geranylgeranyl reductase, plant/prokaryotic
IPR011774	8.916E-04	1.400	0.213	Geranylgeranyl reductase, plant/cyanobacteria
IPR011777	8.916E-04	1.400	0.213	Geranylgeranyl reductase family
IPR005706	9.673E-04	1.175	0.288	Ribosomal protein S2, bacteria/mitochondria/plastid
IPR011545	9.749E-04	0.435	1.825	DEAD/DEAH box helicase domain
IPR012748	9.816E-04	1.833	0.128	Nitrite reductase [NAD(P)H] small subunit, NirD
IPR014001	9.965E-04	0.390	2.199	Helicase superfamily 1/2, ATP-binding domain
IPR001054	1.020E-03	1.062	0.342	Adenylyl cyclase class-3/4/guanylyl cyclase
IPR002073	1.025E-03	1.102	0.320	3'5'-cyclic nucleotide phosphodiesterase, catalytic domain
IPR003029	1.026E-03	0.830	0.534	S1 domain
IPR012756	1.182E-03	1.318	0.224	DNA-directed RNA polymerase, subunit beta''
IPR020547	1.192E-03	1.985	0.107	ATP synthase delta/epsilon subunit, C-terminal domain
IPR029787	1.219E-03	1.046	0.342	Nucleotide cyclase
IPR001650	1.317E-03	0.384	2.156	Helicase, C-terminal
IPR022967	1.327E-03	0.813	0.534	RNA-binding domain, S1
IPR022643	1.360E-03	1.570	0.160	Orn/DAP/Arg decarboxylase 2, C-terminal
IPR022644	1.360E-03	1.570	0.160	Orn/DAP/Arg decarboxylase 2, N-terminal
IPR014102	1.478E-03	1.685	0.139	Phytoene desaturase
IPR007148	1.525E-03	1.448	0.181	Small-subunit processome, Utp12
IPR020846	1.690E-03	0.542	1.089	Major facilitator superfamily domain
IPR007034	1.833E-03	1.382	0.192	Ribosome biogenesis protein BMS1/TSR1, C-terminal

IPR012948	1.833E-03	1.382	0.192	AARP2CN
IPR009006	1.916E-03	1.522	0.160	Alanine racemase/group IV decarboxylase, C-terminal
IPR023179	2.104E-03	1.222	0.235	GTP-binding protein, orthogonal bundle domain
IPR000232	2.345E-03	1.155	0.256	Heat shock factor (HSF)-type, DNA-binding
IPR004693	2.443E-03	1.440	0.171	Silicon transporter
IPR012137	4.120E-03	1.804	0.107	Nitrate reductase NADH dependent
IPR002937	4.606E-03	1.002	0.299	Amine oxidase
IPR018130	4.706E-03	0.860	0.395	Ribosomal protein S2, conserved site
IPR023591	4.706E-03	0.860	0.395	Ribosomal protein S2, flavodoxin-like domain
IPR001138	4.777E-03	2.347	0.064	Zn(2)-C6 fungal-type DNA-binding domain
IPR000511	4.856E-03	2.570	0.053	Cytochrome c/c1 haem-lyase
IPR003728	4.856E-03	2.570	0.053	Ribosome maturation factor RimP
IPR004154	5.558E-03	0.857	0.384	Anticodon-binding
IPR009025	5.565E-03	1.122	0.235	DNA-directed RNA polymerase, RBP11-like dimerisation domain
IPR011262	5.576E-03	1.155	0.224	DNA-directed RNA polymerase, insert domain
IPR011263	5.576E-03	1.155	0.224	DNA-directed RNA polymerase, RpoA/D/Rpb3-type
IPR002146	5.661E-03	1.300	0.181	ATPase, F0 complex, subunit B/B', bacterial/chloroplast
IPR002033	5.717E-03	1.570	0.128	Sec-independent periplasmic protein translocase TatC
IPR005717	6.136E-03	1.833	0.096	Ribosomal protein S7, bacterial/organellar-type
IPR013816	6.338E-03	0.560	0.822	ATP-grasp fold, subdomain 2
IPR030378	6.518E-03	1.164	0.213	Circularly permuted (CP)-type guanine nucleotide-binding (G) domain
IPR002938	6.902E-03	0.863	0.363	FAD-binding domain
IPR011701	6.976E-03	0.685	0.555	Major facilitator superfamily
IPR023753	7.049E-03	0.384	1.633	FAD/NAD(P)-binding domain
IPR008333	8.271E-03	1.137	0.213	Oxidoreductase, FAD-binding domain
IPR011060	8.360E-03	0.821	0.384	Ribulose-phosphate binding barrel
IPR011761	8.566E-03	0.580	0.726	ATP-grasp fold
IPR003607	9.285E-03	0.950	0.288	HD/PDEase domain
IPR010073	9.304E-03	1.570	0.117	Phosphoribosylformylglycinamidine synthase
IPR022653	9.603E-03	1.869	0.085	Orn/DAP/Arg decarboxylase 2, pyridoxal-phosphate binding site
IPR022657	9.603E-03	1.869	0.085	Orn/DAP/Arg decarboxylase 2, conserved site
IPR013005	9.694E-03	1.412	0.139	50S ribosomal protein uL4
IPR010004	9.732E-03	1.740	0.096	Uncharacterised protein family Ycf66
IPR002986	9.738E-03	1.644	0.107	Diaminopimelate decarboxylase, LysA
IPR007109	9.809E-03	1.110	0.213	Brix domain
IPR001865	9.829E-03	0.816	0.374	Ribosomal protein S2

SUPPLEMENTARY REFERENCES

1. O'Leary, N.A., Wright, M.W., Brister, J.R., Ciufo, S., Haddad, D., McVeigh, R., Rajput, B., Robbertse, B., Smith-White, B., Ako-Adjei, D. *et al.* (2016) Reference sequence (RefSeq) database at NCBI: current status, taxonomic expansion, and functional annotation. *Nucleic acids research*, **44**, D733-745.
2. Menzel, P., Ng, K.L. and Krogh, A. (2016) Fast and sensitive taxonomic classification for metagenomics with Kaiju. *Nature communications*, **7**, 11257.
3. Ye, S.H., Siddle, K.J., Park, D.J. and Sabeti, P.C. (2019) Benchmarking Metagenomics Tools for Taxonomic Classification. *Cell*, **178**, 779-794.
4. Huntley, R.P., Sawford, T., Mutowo-Meullenet, P., Shypitsyna, A., Bonilla, C., Martin, M.J. and O'Donovan, C. (2015) The GOA database: gene Ontology annotation updates for 2015. *Nucleic acids research*, **43**, D1057-1063.
5. Zerbino, D.R., Achuthan, P., Akanni, W., Amode, M.R., Barrell, D., Bhai, J., Billis, K., Cummins, C., Gall, A., Giron, C.G. *et al.* (2018) Ensembl 2018. *Nucleic acids research*, **46**, D754-D761.
6. Kersey, P.J., Allen, J.E., Allot, A., Barba, M., Boddu, S., Bolt, B.J., Carvalho-Silva, D., Christensen, M., Davis, P., Grabmueller, C. *et al.* (2018) Ensembl Genomes 2018: an integrated omics infrastructure for non-vertebrate species. *Nucleic acids research*, **46**, D802-D808.
7. UniProt, C. (2021) UniProt: the universal protein knowledgebase in 2021. *Nucleic acids research*, **49**, D480-D489.
8. Friedberg, I. and Radivojac, P. (2017) Community-Wide Evaluation of Computational Function Prediction. *Methods in molecular biology (Clifton, N.J.)*, **1446**, 133-146.
9. Seppey, M., Manni, M. and Zdobnov, E.M. (2019) BUSCO: Assessing Genome Assembly and Annotation Completeness. *Methods in molecular biology (Clifton, N.J.)*, **1962**, 227-245.
